# Tumor-reactive immune cells protect against metastatic tumor and induce immunoediting of indolent but not quiescent tumor cells

**DOI:** 10.1189/jlb.5A1215-580R

**Published:** 2016-02-29

**Authors:** Kyle K. Payne, Rebecca C. Keim, Laura Graham, Michael O. Idowu, Wen Wan, Xiang-Yang Wang, Amir A. Toor, Harry D. Bear, Masoud H. Manjili

**Affiliations:** * Department of Microbiology and Immunology, Virginia Commonwealth University School of Medicine, Richmond, Virginia, USA;; † Massey Cancer Center, Virginia Commonwealth University School of Medicine, Richmond, Virginia, USA;; § Department of Surgery, Virginia Commonwealth University School of Medicine, Richmond, Virginia, USA;; ¶ Department of Pathology, Virginia Commonwealth University School of Medicine, Richmond, Virginia, USA;; ‖ Department of Biostatistics, Virginia Commonwealth University School of Medicine, Richmond, Virginia, USA;; # Department of Human and Molecular Genetics, Virginia Commonwealth University School of Medicine, Richmond, Virginia, USA;; ** Department of Internal Medicine, Virginia Commonwealth University School of Medicine, Richmond, Virginia, USA; and; ‡ The Wistar Institute, Philadelphia, Pennsylvania, USA

**Keywords:** adoptive immunotherapy, dormancy, escape, relapse, breast cancer

## Abstract

Two major barriers to cancer immunotherapy include tumor-induced immune suppression mediated by myeloid-derived suppressor cells and poor immunogenicity of the tumor-expressing self-antigens. To overcome these barriers, we reprogrammed tumor-immune cell cross-talk by combined use of decitabine and adoptive immunotherapy, containing tumor-sensitized T cells and CD25^+^ NKT cells. Decitabine functioned to induce the expression of highly immunogenic cancer testis antigens in the tumor, while also reducing the frequency of myeloid-derived suppressor cells and the presence of CD25^+^ NKT cells rendered T cells, resistant to remaining myeloid-derived suppressor cells. This combinatorial therapy significantly prolonged survival of animals bearing metastatic tumor cells. Adoptive immunotherapy also induced tumor immunoediting, resulting in tumor escape and associated disease-related mortality. To identify a tumor target that is incapable of escape from the immune response, we used dormant tumor cells. We used Adriamycin chemotherapy or radiation therapy, which simultaneously induce tumor cell death and tumor dormancy. Resultant dormant cells became refractory to additional doses of Adriamycin or radiation therapy, but they remained sensitive to tumor-reactive immune cells. Importantly, we discovered that dormant tumor cells contained indolent cells that expressed low levels of Ki67 and quiescent cells that were Ki67 negative. Whereas the former were prone to tumor immunoediting and escape, the latter did not demonstrate immunoediting. Our results suggest that immunotherapy could be highly effective against quiescent dormant tumor cells. The challenge is to develop combinatorial therapies that could establish a quiescent type of tumor dormancy, which would be the best target for immunotherapy.

## Abstract (precis)

Quiescent, but not indolent, dormant tumor cells are resistant to immunoediting, and best targets for immunotherapy of cancer.

## Introduction

MDSCs are key cellular suppressors of anti-tumor immune responses in breast cancer patients. Tumor-derived factors drive the accumulation of MDSCs in the bone marrow and secondary lymphoid organs and at the site of the tumor, thereby inhibiting the efficacy of cellular immunotherapy against established tumors. A number of strategies have been used to enhance immunotherapy of cancer by overcoming MDSCs. These strategies fall into 3 major categories that include MDSC deactivation, depletion of MDSCs, or conversion of MDSCs to APCs [1, 2]. The latter approach identified NKT cells as a key facilitator in promoting MDSC maturation into mature myeloid cells with anti-tumor immune stimulatory function. Therefore, it was suggested that the term M_regs_ better represents the plasticity of these cells, rather than MDSCs [3]. With the use of PBMCs of patients with early-stage breast cancer, we demonstrated previously that an optimal frequency of CD25^+^ NKT cells within reprogrammed immune cells, cultured in the presence of MDSCs/M_regs_, induced them to lose/down-regulate CD11b, which was associated with HLA-DR up-regulation. Such phenotypic modulation was shown to promote anti-human epidermal growth factor receptor 2/neu immune responses in vitro [4]. Therefore, inclusion of CD25^+^ NKT cells in AIT should enhance the anti-tumor efficacy of adoptively transferred T cells by modulating MDSCs/M_regs_ to become immunostimulatory instead of immunosuppressive.

Another barrier to successful cancer immunotherapy is that human cancers are usually poorly immunogenic, with the exception of melanoma. Therefore, the enhancement of the immunogenicity of tumor cells could make them better targets for immunotherapy. On the other hand, tumor immunoediting, such as loss of tumor antigens and engagement of the PD-1/PD-L1 pathway, is likely to occur in the face of robust anti-tumor immune responses. Therefore, the overcoming of tumor immunoediting and escape remains a major challenge for effective anti-cancer immunotherapies. To this end, it is critical to determine how tumors may or may not be prone to immunoediting and escape and how this tendency can be altered.

To address these challenges, we sought to modulate tumor-immune cross-talk by using reprogrammed T cells and NKT cells along with Dec. AIT, with reprogrammed, tumor-sensitized T cells and CD25^+^ NKT cells, is expected to overcome MDSCs and establish memory responses [5], whereas Dec is expected to render tumor cells highly immunogenic by the induction of the expression of CTAs [6, 7]. Dec is an epigenetic therapy for acute myeloid leukemia, which may also inhibit the suppressive function of MDSCs [8]. We evaluated this combinatorial therapy against established primary tumors and against experimental metastasis. Furthermore, we identified 2 types of tumor dormancy, which included indolent dormancy characterized by Ki67^+^/low and quiescent dormancy characterized by Ki67^−^. We demonstrated that quiescent, but not indolent, dormant tumor cells were resistant to immunoediting.

## MATERIALS AND METHODS

### Mouse model

FVBN202 transgenic female mice (The Jackson Laboratory, Bar Harbor, ME, USA) were used. These mice overexpress the nonmutated, nonactivated rat neu transgene under the regulation of the mouse mammary tumor virus promoter [9]. These mice develop premalignant mammary hyperplasia similar to ductal carcinoma in situ before the development of spontaneous carcinoma [10]. These studies have been reviewed and approved by the Institutional Animal Care and Use Committee at Virginia Commonwealth University.

### Tumor cell lines

The neu-overexpressing MMC cell line was established from spontaneous mammary tumors harvested from FVBN202 mice [11]. Tumor cells were maintained in RPMI 1640, supplemented with 10% FBS.

### Ex vivo reprogramming and expansion of splenocytes

The reprogramming of tumor-sensitized immune cells was performed as described previously by our group [5]. In brief, FVBN202 transgenic mice were inoculated in the mammary fat pad with 3 × 10^6^ MMC cells. Tumor growth was monitored by digital caliper, and tumor volumes were calculated by *v* = (*L* × *W*^2^)/2, where *v* is volume, *L* is length, and *W* is width. As described previously [11], splenocytes were harvested 21–25 d after tumor challenge, when the tumor had reached ≥1000 mm^3^. Splenocytes were then cultured in complete medium [RPMI 1640, supplemented with 10% FBS, l-glutamine (2 mM), 100 U/ml penicillin, and 100 μg/ml streptomycin] and were stimulated with Bryostatin 1 (2 nM; Sigma-Aldrich, St. Louis, MO, USA), ionomycin (1 μM; Calbiochem, EMD Millipore, Billerica, MA, USA), and 80 U/ml/10^6^ cells of IL-2 (PeproTech, Rocky Hill, NJ, USA) for 16–18 h. Lymphocytes were then washed thrice and cultured at 10^6^ cells/ml in complete medium with IL-7 and IL-15 (20 ng/ml each cytokine; PeproTech). After 24 h, 20 U/ml IL-2 was added to the complete medium. The following day, the cells were washed and cultured at 10^6^ cells/ml in complete medium with 40 U/ml IL-2. After 48 h, cells were washed and cultured at 10^6^ cells/ml in complete medium with 40 U/ml IL-2. Twenty-four hours later, lymphocytes were again washed and cultured at 10^6^ cells/ml in complete medium with 40 U/ml IL-2. Lymphocytes were harvested 24 h later on the sixth day and were then used for in vitro studies or in vivo for AIT.

### Adoptive cellular immunotherapy

Twenty-four hours before AIT, FVBN202 mice were injected i.p. with CYP (100 mg/kg) to induce lymphopenia. Individual groups of mice were challenged i.d. in the mammary gland region, with 3 × 10^6^ MMC cells, or i.v. with 10^6^ MMC. Individual groups of mice then received reprogrammed splenocytes i.v. at a dose of 70 × 10^6^/mouse, 3 d after tumor challenge when the tumor became palpable (50–70 mm^3^) or on the day of the i.v. tumor injection. Untreated tumor-bearing mice served as control.

### In vitro and in vivo induction of CTA expression in MMC cells and cDNA synthesis

MMC cells (3 × 10^6^ cells/3 ml) were cultured in the presence of 3 µM Dec (Sigma-Aldrich) for 72 h. Medium was then removed, and cells were washed with sterile PBS and then treated with TRIzol (Life Technologies, Thermo Fisher Scientific, Grand Island, NY, USA), per the manufacturer’s instructions. In vivo, FVBN202 mice, bearing primary tumor ≥1000 mm^3^, were injected i.p. with a high-dose Dec (2.5 mg/kg), once daily for 5 d. Mice were euthanized, and tumors were harvested 3 d later, minced, and then treated with TRIzol, per the manufacturer’s instructions. Contaminant DNA was then removed by DNase I digestion from the in vitro and in vivo specimens; RNA was then purified, followed by cDNA synthesis, as described previously by our group [12].

### Real-time qRT-PCR for the detection of CTA expression

qRT-PCR was performed in triplicate wells using the SensiMix SYBR & Fluorescein Kit, according to the manufacturer’s procedure (Bioline, Taunton, MA, USA), with the CFX96 Real-Time PCR Detection System (Bio-Rad Laboratories, Hercules, CA, USA). qRT-PCR was performed using primers specific for 6 murine CTAs and murine GAPDH. The reaction was initiated by a denaturing period of 10 min at 95°C, followed by 40 cycles of 95°C for 15 min, 60°C for 30 min, and 72°C for 15 min [6, 12]. Relative CTA expression was computed after normalization to GAPDH using the ΔΔ quantification cycle method.

### IFN-γ ELISA

Reprogrammed immune cells were cultured in complete medium with irradiated (140 Gy) MMC cells or irradiated CTA-expressing MMC, induced by Dec treatment in vitro at a 10:1 ratio for 20 h. Supernatants were then collected and stored at −80°C until assayed. IFN-γ was detected using a mouse IFN-γ ELISA kit (BD Biosciences, Franklin Lakes, NJ, USA), according to the manufacturer’s protocol [5].

### Characterization of splenocytes and tumor-infiltrating leukocytes

Spleens and metastatic tumor lesions of FVBN202 mice were harvested when the animals became moribund and were then separately homogenized into a single cell suspension as described previously [11] and below. Splenocytes were then characterized using flow cytometry. Reagents used for flow cytometry include the following: anti-CD16/32 antibody (clone 93), FITC-CD3 (17A2); FITC-CD11b (M1/70); FITC-anti-mouse IgG (Poly4053); PE-GR-1 (RB6-8C5); PE-PD-1 (RMP1-30); PE-CD25 (3C7); PE-Ki67 (16A8); allophycocyanin-CD49b (DX5); allophycocyanin-CD62 ligand (MEL-14); allophycocyanin-Annexin V; PerCP/CY5.5-CD4 (GK1.5); PE/CY7-CD8α (53-6.7); Brilliant Violet 421-PD-L1 (10F.9G2); Brilliant Violet 605-CD45 (30-F11); and PI, all of which were purchased from BioLegend (San Diego, CA, USA). BD Horizon V450-Annexin V and FITC-FVS were purchased from BD Biosciences. Anti-rat neu antibody (anti–c-Erb2/c-Neu; 7.16.4), was purchased from Calbiochem. All reagents were used at the manufacturer’s recommended concentration. Cellular staining was performed as described previously by our group [11] or as recommended by the manufacturer (Ki67, FVS). Multicolor data acquisition was performed using a LSRFortessa X-20 (BD Biosciences). Data were analyzed using FCS Express v4.07 (De Novo Software, Glendale, CA, USA).

### Isolation and characterization of lung metastases

Lungs were harvested from the "Control" and "AIT" groups after animals became moribund. Metastatic lesions were excised individually from the residual lung tissue and minced and digested in Trypsin-EDTA (0.25%; Life Technologies, Thermo Fisher Scientific) overnight at 4°C. The following day, the suspension was incubated at 37°C for 30 min, followed by gentle tissue homogenization to create a cellular suspension. The cell suspension was then washed twice with RPMI supplemented with 10% FBS. Residual RBCs were then lysed using ammonium-chloride-potassium lysing buffer, followed by an additional wash with RPMI 10% FBS. The cell suspension was then placed in cell culture and cultured with RPMI 10% FBS. Adherent metastatic tumor cells were then characterized for the expression of rat neu and PD-L1 using flow cytometry.

### Characterization of metastatic tumor-infiltrating leukocytes

Lungs from each group were harvested, and metastatic lesions were isolated as described above. After tissue digestion of the metastatic lesions and RBC lysis, 10^6^ cells of the suspension were placed in flow tubes and stained for surface molecules as described above. All analysis was performed by gating on viable leukocytes (Annexin V^−^ CD45^+^), thereby discriminating against apoptotic cells and tumor cells.

### Establishment of ex vivo tumor cell dormancy

MMC cells were treated with 3 daily doses of ADR (doxorubicin hydrochloride, 1 µM/d for 2 h; Sigma-Aldrich). Residual, dormant MMC cells remained adherent to tissue-culture flasks, whereas the MMC cells susceptible to ADR therapy became nonadherent and were removed from the culture periodically. Assessment of viability, Ki67 expression, and IFN-γ-induced PD-L1 up-regulation by flow cytometry occurred 3 wk after the final treatment. Likewise, 3 daily doses of RT (2 Gy/d) were also used to establish dormant MMC cells. ADR and RT-induced dormant MMC cells were used in the cytotoxicity assay, 8 d after the final treatment.

### IHC

Sections of formalin-fixed paraffin-embedded tissue from each tumor were stained with H&E to examine the histomorphology. Additional sections are then subsequently immunolabeled using the standard IHC technique, using the avidin-biotin peroxidase system with a purified anti-mouse Ki67 (BioLegend). Sections of lymph nodes were used as the positive control. Nikon Eclipse 80i light microscope was used to examine the H&E and IHC. The most intense labeling regions (hot spots) away from the edge of the tissue were evaluated using IHC-positive tumor cells as numerator and the overall tumor cells as the denominator. Representative images of the H&E and the corresponding hotspots were taken.

### Statistical analysis

Statistical comparisons between groups were made using 1- and 2-tailed Student's *t* test. Time to death in the in vivo survival studies was calculated from baseline to the date of death. Mice were euthanized when they had a weight loss of ≥10%. Kaplan-Meier curves and log-rank tests are used to illustrate time to death and to test the difference between each group. *P* ≤ 0.05 was considered statistically significant.

## RESULTS

### The reprogramming of tumor-immune cross-talk during immunotherapy fails to protect animals from an established primary mammary carcinoma

**Figure 1.**
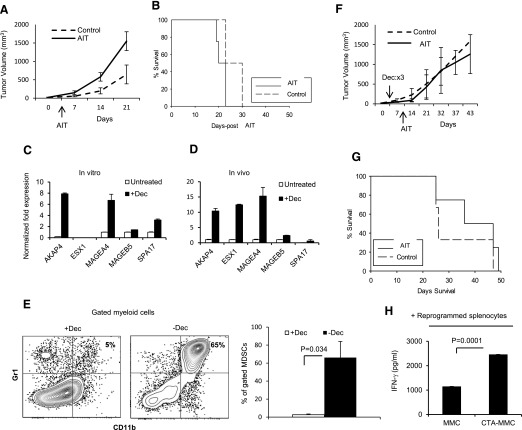
AIT, with or without Dec, fails to induce regression-established mammary carcinoma. (A and B) Animals were challenged with MMC (3 × 10^6^) i.d. in the mammary gland region; upon the tumor reaching 50–70 mm^3^, animals were conditioned with CYP (100 mg/kg). The following day, mice remained untreated (Control; *n* = 4) or received AIT (*n* = 4). (C) MMC tumor cells were cultured with (+Dec) or without Dec (Untreated; 3 mM) for 72 h; RNA was then extracted and converted to cDNA, followed by qRT-PCR, using primers specific for 6 murine CTAs. (D) Tumor-bearing (∼1000 mm^3^ i.d.) FVBN202 mice received 5 injections of Dec, 1/d (+Dec; 2.5 mg/kg; *n* = 1) or remained untreated (*n* = 1); the tumors were harvested 3 d later, and cDNA was generated to quantify CTA expression, which was normalized to GAPDH. AKAP4, A-kinase anchor protein 4; ESX1, ; MAGEA4, melanoma-associated antigen 4; MAGEB5, melanoma-associated antigen B5; SPA17, ESX1, Extraembryonic, spermatogenesis, homeobox 1; SPA17, Sperm Autoantigenic Protein 17. (E) Animals were challenged i.d. with MMC (3 × 10^6^) in the mammary gland region; after tumors reached 50–70 mm^3^, all animals were treated with Dec (every other day for 3 total injections; 2.5 mg/kg, i.p.; *n* = 3) or remained untreated (*n* = 3). Seven days later, mice were euthanized, and MDSCs were analyzed in the spleen. (F and G) Animals were challenged i.d. with MMC (3 × 10^6^) in the mammary gland region; after tumors reached 50–70 mm^3^, all animals were treated with Dec [every day for 3 total injections (×3); 2.5 mg/kg, i.p.]. Two days later, animals were conditioned with CYP (100 mg/kg, i.p.). The following day, mice remained untreated (Control; *n* = 3) or received AIT (*n* = 4), derived from a CTA^+^ tumor-bearing donor. (H) MMC cells remained untreated (MMC) or were treated with Dec (3 mM; 72 h) to induce CTA expression (CTA-MMC). Tumor cells were then cocultured with reprogrammed splenocytes (1:10) for 20 h. IFN-γ was detected in the supernatant by ELISA. Data represent means ± sem of duplicate wells.

We have reported previously that AIT, using reprogrammed T cells and NKT cells in a prophylactic setting, protected animals against primary tumors and recall tumor challenge. This protection was associated with the presence of memory T cells, and CD25^+^ NKT cells that rendered T cells resistant to MDSC-mediated suppression [5]. Here, we sought to determine whether AIT as a single therapy can protect animals against established primary mammary carcinoma by overcoming MDSCs. FVBN202 mice bearing primary tumors received AIT using reprogrammed T cells and CD25^+^ NKT cells when the tumor had reached 50–70 mm^3^ or remained untreated. As shown in **Fig. 1A** and **B**, AIT alone did not slow the rate of tumor growth (Fig. 1A) or improve overall survival (Fig. 1B) in recipient mice compared with untreated control mice. Then, we combined AIT with epigenetic modulation of tumor cells in vivo to enhance immunogenicity of tumor cells, as well as eliminate MDSCs. To do so, tumor-bearing animals received Dec before AIT. Use of Dec induced the expression of a panel of CTAs in tumor cells (Fig. 1C and D) and resulted in the elimination of MDSCs (Fig. 1E; *P* = 0.034). However, AIT still failed to protect animals from established primary tumors when compared with Dec alone (Fig. 1F and G). This failure was observed in spite of successful reprogramming of tumor-sensitized immune cells (Supplemental Fig. 1) and their enhanced reactivity against CTA-expressing MMC (Fig. 1H; *P* = 0.0001).

### The reprogramming of tumor-immune cross-talk during immunotherapy prolongs survival of animals bearing metastatic tumor cells in their circulation

**Figure 2.**
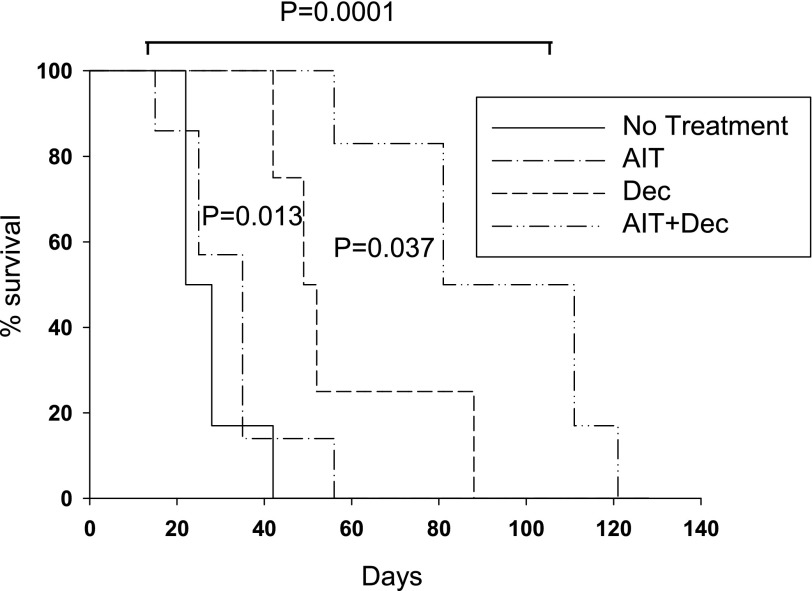
Combined use of Dec and AIT prolongs survival of animals and induces tumor immune editing. FVBN202 mice were challenged with 1 × 10^6^ MMC cells i.v. Mice then remained untreated (Control; *n* = 4), received AIT (*n* = 7) on the same day as tumor challenge, received Dec (Dec; *n* = 4; 5 daily doses beginning on d 3 after tumor challenge), or received Dec and AIT (AIT + Dec; *n* = 6; AIT on the day of tumor challenge, followed by Dec beginning on d 3).

We have reported previously that administration of AIT along with another epigenetic modulator, Aza, was effective in prolonging survival of patients with multiple myeloma when treatment was delivered during minimal residual disease to prevent advanced stage disease [6]. Therefore, we sought to take a similar approach in our experimental model of breast cancer by administrating AIT and Dec when tumor cells were present in the circulation and before establishing lung metastasis. AIT alone had a marginal impact on the survival of animals, whereas Dec alone resulted in prolonging the survival of animals (**Fig. 2**; P = 0.013). AIT + Dec was the most effective therapy that resulted in prolonging the survival of animals compared with the control group or Dec alone (Fig. 2; *P* = 0.0001 and *P* = 0.037, respectively). However, all animals eventually succumbed to metastatic tumors in the lungs.

### Immunotherapy induces tumor immunoediting and escape in proliferating tumor cells and indolent dormant cells but not in quiescent dormant cells

**Figure 3.**
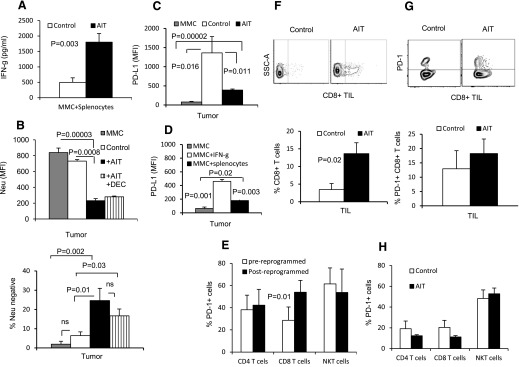
AIT promotes immunoediting of lung metastatic lesions. (A) Splenocytes were harvested from untreated mice (Control; *n* = 5) and AIT recipients (*n* = 3) and cultured in the presence of MMC cells (10:1) for 20 h. Supernatants were collected and subjected to IFN-γ ELISA. (B) Metastatic lesions in the lung of FVBN202 mice that remained untreated (Control; *n* = 3), AIT recipients (*n* = 6), and AIT + Dec recipients (*n* = 4) were harvested when mice became moribund. Tumor lesions were digested and analyzed. MFI of neu and frequency of neu loss (B) and MFI of PD-L1 (C) were then quantified using flow cytometry using MMC cell line (MMC) as an in vitro control. (D) MMC cells were cultured with IFN-γ or splenocytes of tumor-bearing mice, and PD-L1 was detected after 16–20 h (*n* = 2–3). (E) Spleens of FVBN202 mice bearing primary mammary carcinoma (*n* = 4) were harvested after tumors were ≥1000 mm^3^. PD-1 expression was then quantified on the splenocytes, pre- and postreprogramming. (F and G) Metastatic lesions in the lung of FVBN202 mice that remained untreated (Control) and AIT recipients were harvested when mice became moribund. (F) The frequency of CD8^+^ T cell infiltration metastatic lesion in the lung of control mice and the AIT group (*n* = 3) was determined on gated CD3^+^ cells. SSC-A, Side-scatter-area. (G) Expression of PD-1 was determined on CD3^+^CD8^+^ cells (Control, *n* = 1; AIT, *n* = 3) by gating on CD45^+^ viable leukocytes. (H) Spleens of FVBN202 mice that had received AIT (*n* = 4) or remained untreated (*n* = 3) and were i.v. challenged with MMC were analyzed by flow cytometry after tumor-bearing mice became moribund to quantify PD-1 expression. Data represent means ± sem.

To determine whether reprogrammed memory T cells were maintained in vivo, splenocytes of AIT recipients were collected when mice became moribund and cultured with MMC tumor cells. As shown in **Fig. 3A**, tumor-reactive IFN-γ production by endogenous splenic T cells from the AIT group was greater than that produced by T cells from the control group (*P* = 0.003). To determine the impact of treatments on tumor immunoediting and escape, T cells and tumor cells in the tumor-microenvironment of the lung were analyzed. Metastatic tumor lesions were isolated from the lung at the end of the trial and analyzed for the expression of the tumor antigen, neu, and PD-L1. The tumor lesions isolated from the AIT group showed down-regulation of neu antigen on the tumor cells compared with control MMC tumor cells and the lesions isolated from the control group (Fig. 3B, upper; *P* = 0.00003 and *P* = 0.0008, respectively). The AIT + Dec group showed similar trends as the AIT group. Additionally, 25% of MMC cells isolated from metastatic tumor lesions of the AIT group demonstrated total loss of neu expression compared with control MMC tumor cells and the lesions isolated from control group (Fig. 3B, lower; *P* = 0.002 and *P* = 0.01, respectively). Again, the AIT + Dec group showed similar trends as the AIT group. This suggests that metastatic MMC cells may eventually escape detection from neu-specific cellular immunity. Metastatic tumors of the control group that received no treatment did not show down-regulation or loss of neu antigen (Fig. 3B). As AIT was the major factor in neu loss/down-regulation, and the AIT-Dec group showed a similar trend, we looked at the expression of tumor PD-L1 expression in the AIT group. Interestingly, metastatic tumor cells from the control group had higher expression of PD-L1 in the tumor compared with the metastatic tumor cells from the AIT group or control MMC cells (Fig. 3C; MFI: 1360 vs. 390 ± sem; *P* = 0.011). A similar trend was observed when MMC cells were cultured with IFN-γ or splenocytes of tumor-bearing animals (Fig. 3D).

The immune-suppressive function of PD-L1 requires engagement with PD-1, which renders immune cells unresponsive [13, 14]. Importantly, 40–50% of reprogrammed T cells and NKT cells that were used for AIT expressed PD-1 (Fig. 3E), but only CD8^+^ T cells were observed to up-regulate PD-1 as a result of reprogramming (Fig. 3E; *P* = 0.01). Therefore, we also analyzed tumor-infiltrating T cells for PD-1 expression to determine the potential for the PD-1/PD-L1 axis to mediate T cell suppression within the tumor site. Interestingly, as seen in Fig. 3F, tumor infiltration of CD8^+^ T cells into the tumor bed was greater in mice receiving AIT compared with untreated mice (14% vs. 3%, respectively; *P* = 0.02). However, expression of PD-1 on tumor-infiltrating CD8^+^ T cells did not significantly increase following AIT compared with the control group (Fig. 3G). We did not observe CD4^+^ T cell infiltration into the tumor lesions (data not shown). Splenic T cells and NKT cells that were isolated from the AIT and control groups when animals became moribund also expressed PD-1, although there was no statistical difference between the groups (Fig. 3H; 10% of T cells and 50% of NKT cells). Taken together, these data suggest that although AIT promotes the infiltration of CD8^+^ T cells, the highly proliferative nature of the metastatic tumors may evade such anti-tumor immune responses by emerging with reduced expression of the tumor antigen, neu. Metastatic tumor of the Dec group also showed down-regulation of neu antigen (Supplemental Fig. 2A, left; MFI: 442 vs. 202 ± sem), as well as total loss of neu antigen in 36% of tumor cells compared with the control MMC cell line containing a residual 5% of neu-negative cells (Supplemental Fig. 2A, right). As the control group did not show neu loss or down-regulation, but the AIT group did, we sought to determine whether neu loss or down-regulation in animals who received Dec could result from the contribution of an endogenous T cell response induced by Dec, which induces the expression of CTAs and therefore, could function as an in situ vaccination by eliciting endogenous T cell responses. To determine the contribution of Dec in neu antigen loss or down-regulation, we performed in vitro studies by treatment of MMC with Dec alone, where the endogenous immune response did not have any contribution. Dec treatment resulted in the quantitative down-regulation of neu expression but did not induce total neu loss (Supplemental Fig. 2B; *P* = 0.008). IFN-γ induced down-regulation of neu (Supplemental Fig. 2C; *P* = 0.002), and Dec did not recover neu expression. Thus, we then began to question whether residual tumor cells that remain after conventional cytotoxic therapy, which are generally dormant, also use similar escape mechanisms or if they were perhaps more sensitive to immune-mediated elimination.

**Figure 4.**
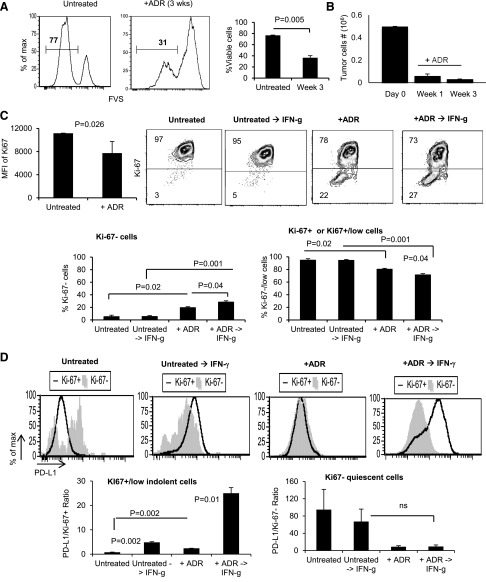
ADR treatment results in the emergence of indolent and quiescent tumor dormancy. (A) MMC tumor cells were treated with 3 daily doses of ADR (1 µM for 2 h) and then remained untreated for 3 wk. The frequency of viable MMC cells was determined by quantifying FVS^−^ cells using flow cytometry. (B) At wk 1 and 3 post-treatment, adherent and viable tumor cells were counted by trypan blue exclusion. (C and D) MMC cells were treated for 3 consecutive d with ADR (1 µM, 2 h) or left untreated. Three weeks later, ADR-treated and untreated MMC cells were stimulated with IFN-γ (50 ng/ml) for 12–16 h to induce the expression of PD-L1. (C) Emergence of Ki67 was determined in control MMC cells (Untreated), as well as ADR-treated cells (+ADR) ± IFN-γ stimulation. (D) The expression of PD-L1/cell was calculated by dividing PD-L1 MFI by the frequency of Ki67^−^ cells in ADR-treated and untreated MMC cells ± IFN-γ stimulation. Data represent 3 independent experiments and means ± sem.

To determine whether dormant tumor cells were resistant to immunoediting and escape, MMC tumor cells were treated with ADR to establish tumor dormancy ex vivo. Dormant tumor cells were then treated with a product of anti-tumor T cell responses—IFN-γ—to determine sensitivity of different dormant cells, quiescent and indolent, to immunoediting. We looked at the expression of PD-L1, as this is the most immediate immunoediting change that occurs as a result of IFN-γ treatment. A clinically relevant proliferation marker, Ki67, along with a viability marker (FVS), was used to detect FVS^−^ viable, indolent tumor cells (Ki67^+^/low) and quiescent tumor cells (Ki67^−^). As shown in **Fig. 4A**, ADR induced apoptosis in the majority of MMC cells, such that by 3 wk after treatment, the number of FVS^−^ viable MMC cells was reduced from 77% to 31% (*P* = 0.005). The remaining residual viable tumor cells that escaped ADR-induced apoptosis entered a state of dormancy, as there was no significant increase in the number of tumor cells between 1 and 3 wk after completion of ADR treatment (Fig. 4B). To determine if dormant tumor cells could exploit immune escape mechanisms, we established ADR-induced tumor dormancy, followed by treatment of dormant MMC cells with IFN-γ, 3 wk after the completion of ADR treatment to provoke PD-L1 expression [15]. We evaluated the expression of PD-L1 on viable proliferating control MMC cells (Ki67^+^), without (Untreated) and with IFN-γ (Untreated → IFN-γ) treatment, as well as on viable dormant tumor cells without (+ADR) and with IFN-γ (+ADR → IFN-γ) treatment. As shown in Fig. 4C, left, ADR-treated MMC showed a significant shift from Ki67^+^ toward Ki67^+^/low cells (*P* = 0.026), indicative of indolent tumor dormancy. ADR or ADR → IFN-γ treatment also resulted in a shift from Ki67^+^ toward Ki67^−^ quiescent cells, as shown by an increased frequency of Ki67^−^ MMC (Fig. 4C; *P* = 0.02 and *P* = 0.001, respectively) and a decreased frequency of Ki67^+^ MMC cells (Fig. 4C; *P* = 0.02 and *P* = 0.001, respectively). IFN-γ treatment induced up-regulation of PD-L1 on Ki67^+^/low indolent MMC (Fig. 4D, left; *P* = 0.002 and *P* = 0.01). Interestingly, Ki67^−^ control MMC cells and Ki67^−^ quiescent MMC cells did not up-regulate PD-L1 in the presence of IFN-γ (Fig. 4D, right).

### Dormant MMC cells established by ADR or RT become resistant to higher doses of chemotherapy or RT but remain sensitive to immunotherapy

**Figure 5.**
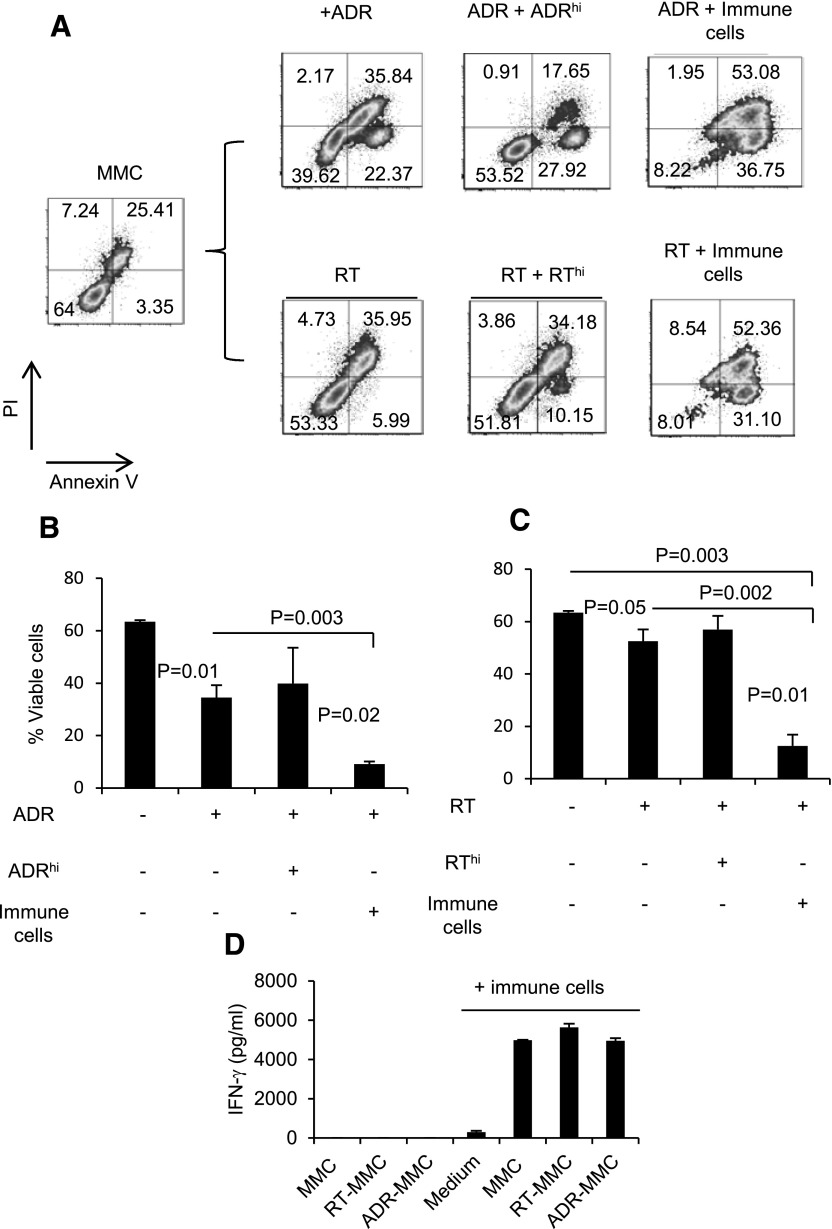
Immunotherapy displays cytotoxic function against treatment refractory dormant tumor cells in vitro. (A) MMC cells (*n* = 3) treated with ADR (1 µM, 2 h) or 2 Gy RT (RT-treated MMC) for 3 consecutive d and remained in culture for 8 d total to establish tumor cell dormancy in vitro. (B) On d 8, these dormant tumor cells were treated with a high-dose ADR (1 µM, 24 h; ADR-treated MMC + ADR^hi^) or reprogrammed immune cells (ADR-treated MMC + immune cells; ADR-treated MMC + immune cells). Two days later, cells were stained with Annexin V/PI and analyzed by flow cytometry. Data represent 3 biologic repeats and means ± sem. (C) On d 8, these dormant tumor cells were treated with 18 Gy RT (RT-treated MMC + RT^hi^) or reprogrammed immune cells (RT-treated MMC + immune cells). Two days later, cells were stained with Annexin V/PI and analyzed by flow cytometry. (D) MMC tumor cells or dormant MMC cells (RT-MMC, ADR-MMC) were cultured in the absence or presence of the reprogrammed immune cells in a 10:1 ratio for 24 h. Control immune cells were cultured alone (Medium). IFN-γ release was detected in the supernatant using ELISA. Data represent 2 biologic repeats and means ± sem.

ADR chemotherapy usually induces tumor dormancy, which could lead to tumor recurrence. To determine the direct effect of ADR on tumor dormancy, we performed ex vivo experiments. ADR treatment increased the proportion of Ki67^−^ tumor cells, which lasted for 3 wk. This trend was associated with reduced viability, 3 wk after treatment, which improved 6 wk after treatment (Supplemental Fig. 3; 77% > 31% > 50%). To determine whether dormant MMC cells established by ADR treatment remain sensitive to tumor-reactive immune cells, dormancy was established by treating MMC with 3 daily doses of ADR (1 µM/d for 2 h; Supplemental Fig. 4A); 8 days after the final treatment, MMC cells received a high dose of ADR (1 µM for 24 h) or were cultured with tumor-reactive immune cells for 48 h. ADR treatment induced apoptosis in MMC cells (**Fig. 5A** and **B**; *P* = 0.01). Tumor cells that survived apoptosis became chemo refractory, such that additional ADR treatment at a higher dose (1 µM for 24 h) did not induce cell death (Fig. 5A and B; average 40% vs. 54%). However, they remained sensitive to tumor-reactive immune cells. In the presence of tumor-reactive immune cells, the frequency of viable ADR-treated dormant MMC dropped from 40% to 8% (Fig. 5A and B; *P* = 0.003). In fact, lymphocytes were more effective than a high dose of chemotherapy in inducing apoptosis in dormant MMC (Fig. 5A and B; *P* = 0.02). We also established dormant MMC by 3 daily doses of RT (2 Gy/d); again, surviving dormant cells became refractory to RT. An additional RT at a higher dose (18 Gy) did not markedly decrease the frequency of viable tumor cells (Fig. 5B and C; 53% vs. 52%). However, RT refractory MMC cells remained sensitive to tumor-reactive lymphocytes as the viability dropped from 53% to 8% (Fig. 5B and C; *P* = 0.002). In recapitulating our results with chemotherapy-induced tumor cell dormancy, tumor-reactive immune cells were more effective than high-dose RT at inducing apoptosis in dormant MMC (Fig. 5B and C; *P* = 0.01). To determine whether higher levels of apoptosis in dormant tumor cells were a result of their greater sensitivity to immune cells rather than a higher reactivity of the immune cells, IFN-γ ELISA was performed using reprogrammed immune cells cultured with MMC tumor cells or ADR- and RT-induced dormant MMC cells. As shown in Fig. 5D, tumor-reactive immune cells produced comparable levels of IFN-γ upon stimulation with MMC or dormant MMC (RT-MMC, ADR-MMC). To determine the establishment of Ki67^−^ quiescent and Ki67^+^/low indolent tumor cells, experimental animals bearing primary MMC were treated with ADR or remained untreated. Animals treated with ADR exhibited suppression of tumor growth (Supplemental Fig. 4B). Tumors of the ADR group showed a shift from Ki67^+^ toward Ki67^+^/low indolent and Ki67^−^ quiescent cells (Supplemental Fig. 4C).

## DISCUSSION

We developed an experimental metastatic mouse model by i.v. injection of highly proliferative MMC cells to FVBN202 mice. Animals in this model became moribund within 20-40 d and presented with lung metastases upon macroscopic inspection. This model represents the onset of advanced stage disease. We demonstrated that concurrent use of Dec with AIT using reprogrammed CD25^+^ NKT and T cells prolonged survival of the experimental animals but failed to eliminate the tumor, as all mice eventually succumbed to metastatic disease in the lungs. Failure of tumor elimination was associated with down-regulation of the tumor antigen, neu, on metastatic tumor cells. Studies involving AIT without Dec treatment in vivo or Dec alone without immune response in vitro confirmed that total neu antigen loss and down-regulation were mediated by anti-tumor immune responses, whereas Dec alone only had the capacity to induce down-regulation of neu antigen. We have reported previously that treatment of neu-negative tumor cells (antigen-negative variant) with Dec resulted in the induction of neu expression at mRNA but not at protein levels [11]. Likewise, Dec treatment did not overcome IFN-γ-induced down-regulation of neu protein in MMC. Here, we also showed that a high dose of Dec induced down-regulation of the neu protein in vivo. These data suggest that high-dose Dec might have different effects on neu expression during mRNA transcription and protein translation. It was reported that a low dose of Dec could have only a hypomethylating effect when incorporated into DNA, whereas a high-dose Dec could also incorporate into RNA and show different effects [16]. Although Dec or Aza render tumor cells highly immunogenic by inducing the expression of highly immunogenic CTAs, this effect is usually transient in that tumor cells lose CTAs after the cessation of Aza therapy [6].

We hypothesized that targeting dormant but not highly proliferating tumor cells might overcome tumor immunoediting and escape. Therefore, we conducted studies to determine the sensitivity of dormant tumor cells to immunoediting and escape. We demonstrated that ADR treatment induced 2 types of tumor dormancy: 1) an indolent type of dormancy, characterized by the positive/low expression of Ki67; this type of dormancy is maintained through balanced proliferation and death, as these cells keep producing dead cells, whereas the total number of viable cells remains unchanged; and 2) a quiescent type of tumor dormancy that is characterized by lack of Ki67 expression (Ki67^−^); this type of dormancy is maintained through total cessation of proliferation. We demonstrated that proliferating tumor cells, either untreated tumor cells (Ki67^+^) or indolent tumor cells (Ki67^+^/low), were susceptible to immunoediting and escape during cell division, but quiescent tumor cells (Ki67^−^) failed to undergo immunoediting; in fact, they failed to up-regulate PD-L1 in the presence of IFN-γ stimulation. These results suggest that quiescent dormant cells could be the best target for immunotherapy.

It was reported that innate IFN-γ is essential for up-regulation of PD-L1 expression [17]. Intriguingly, an adaptive immune response following AIT resulted in a >3-fold inhibition in the induction of PD-L1 expression on tumor cells compared with the control group, although it was still significantly higher than MMC tumor cells in vitro before challenge. Similar results were obtained when tumor cells were cultured with IFN-γ or lymphocytes of tumor-bearing mice. These data suggest that a T cell-independent inflammatory response, which involves IFN-γ, has a greater impact than T cells on up-regulation of PD-L1. In addition, AIT was associated with a significant inhibition in the induction of tumor PD-L1 compared with no AIT control group, suggesting that the PD-1/PD-L1 axis is more active in tumor-bearing animals in the absence of AIT. To test this, we cocultured reprogrammed tumor-reactive T cells with MMC in the presence or absence of PD-1 blocking antibody; the anti-tumor function of reprogrammed T cells was not affected by PD-1 blockade in vitro (data not shown).

This is important, as reprogrammed T cells and NKT cells that were used for AIT expressed PD-1, and PD-1 expression was sustained after AIT. However, reprogrammed T cells also produce perforin and granzyme B [5], allowing them to induce apoptosis in tumor cells before they begin to up-regulate PD-L1 mediated by IFN-γ. These data suggest that AIT results in a significant inhibition of tumor PD-L1 induction to the levels that might not be suppressive. Blockade of PD-1/PD-L1 by anti-PD-1 antibody did not have any effects on anti-tumor immune responses against MMC in vitro (data not shown). Therefore, prolonged survival in the AIT group could be associated with lower expression of PD-L1 in MMC when tumor cells were present in the circulation compared with the control group when tumors were established in the lungs. Given the high levels of PD-L1 expression on established tumors, administration of AIT as a single agent in a therapeutic setting is likely to fail in curing cancer, as it was evident in our therapeutic protocol (Fig. 1). Administration of AIT in a preventive setting, when tumor cells were in the circulation but before they establish lung metastasis, was highly effective, although animals succumbed to metastatic tumor, as their tumors begin to undergo neu antigen loss. Our data suggest that tumors use numerous mechanisms to change during cell division and escape from immunotherapy. These mechanisms were shown to overcome tumor immune surveillance and reduce the efficacy of immunotherapy [18, 19]. However, and intriguingly, dormant tumor cells that were established by chemotherapy or RT and that became chemo resistant or RT resistant remained sensitive to tumor-reactive immune cells. Our findings are consistent with the reports on the efficacy of AIT in patients with metastatic melanoma using TILs grown in IL-2. AIT, using IL-2-expanded TIL, resulted in tumor regression in 49% of patients [20]. When AIT was combined with total body irradiation, which was implemented to induce lymphopenia, objective responses increased to 72%. Among treated groups, 20% had complete tumor regression and >10 y relapse-free survival [21]. Thus far, of the 34 complete responders in the National Cancer Institute trials, 1 has recurred [22].

The results of this study suggest that administration of immunotherapy in a setting of advanced stage prophylaxis, i.e., after the completion of conventional cancer therapies, when tumor dormancy is established but before distant recurrence of the disease, could effectively target dormant tumor cells and prevent advanced stage disease. On the other hand, the application of immunotherapy to highly proliferative tumors renders the tumors prone to immunoediting and subsequent immunologic escape during cell division [23]. The challenge is to develop combinatorial therapies, i.e., AIT, following the administration of epigenetic modulators or small molecules that could induce cell-cycle arrest and establish a quiescent type of tumor dormancy so as to render dormant tumor cells resistant to immunoediting and escape from immunotherapy. The extension of knowledge gained from our preclinical studies to the clinical setting remains to be determined in patients with early-stage breast cancer or patients with minimal residual disease.

## AUTHORSHIP

K.K.P. and M.H.M. contributed to the study’s conception, design, experimental and analytical performance, and writing of the manuscript. R.C.K., L.G., M.O.I., and W.W. contributed to the study’s experimental and analytical performance and writing of the manuscript. A.A.T., X.-Y.W., and H.D.B. contributed to the study’s conception, analytical performance, and writing of the manuscript,

## Supplementary Material

Supplemental Data

## Abbreviations

ADR: Adriamycin
AIT: adoptive immunotherapy
Aza: azacytidine
CTA: cancer testis antigen
CYP: cyclophosphamide
Dec: decitabine
FVS: fixable viability stain
FVS^−^: FVS-negative
i.d.: intradermally
IHC: immunohistochemistry
i.p.: intraperitoneally
i.v.: intravenously
Ki67^−^: Ki67-negative
Ki67^+^/low: low levels of Ki67
MDSC: myeloid-derived suppressor cell
MFI: mean fluorescence intensity
MMC: mouse mammary carcinoma
M_reg_: myeloid regulatory cell
PD-1: programmed death 1
PD-L1: programmed death ligand 1
PI: propidium iodide
qRT-PCR: quantitative RT-PCR
RT: radiation therapy
TIL: tumor-infiltrating lymphocyte
