# Supplementary material for: Tumor-reactive immune cells protect against metastatic tumor and induce immunoediting of indolent but not quiescent tumor cells
Source: J Leukoc Biol. 2016 Feb 29;100(3):625–35. doi: 10.1189/jlb.5A1215-580R (PMC4982610; doi:10.1189/jlb.5A1215-580R)
Supplement: Supplemental Data [file supp_100_3_625__index.html]

Tumor-reactive immune cells protect against metastatic tumor and induce immunoediting of indolent but not quiescent tumor cells — Tumor-reactive immune cells protect against metastatic tumor and induce immunoediting of indolent but not quiescent tumor cells — Supplemental Data 

# Tumor-reactive immune cells protect against metastatic tumor and induce immunoediting of indolent but not quiescent tumor cells

## Supplemental Data

- Supplemental Data
