# Supplementary material for: Tumor-reactive immune cells protect against metastatic tumor and induce immunoediting of indolent but not quiescent tumor cells
Source: J Leukoc Biol. 2016 Feb 29;100(3):625–35. doi: 10.1189/jlb.5A1215-580R (PMC4982610; doi:10.1189/jlb.5A1215-580R)
Supplement: Supplemental Data [file supp_jlb.5A1215-580R_Supplemental_Figures.pdf]

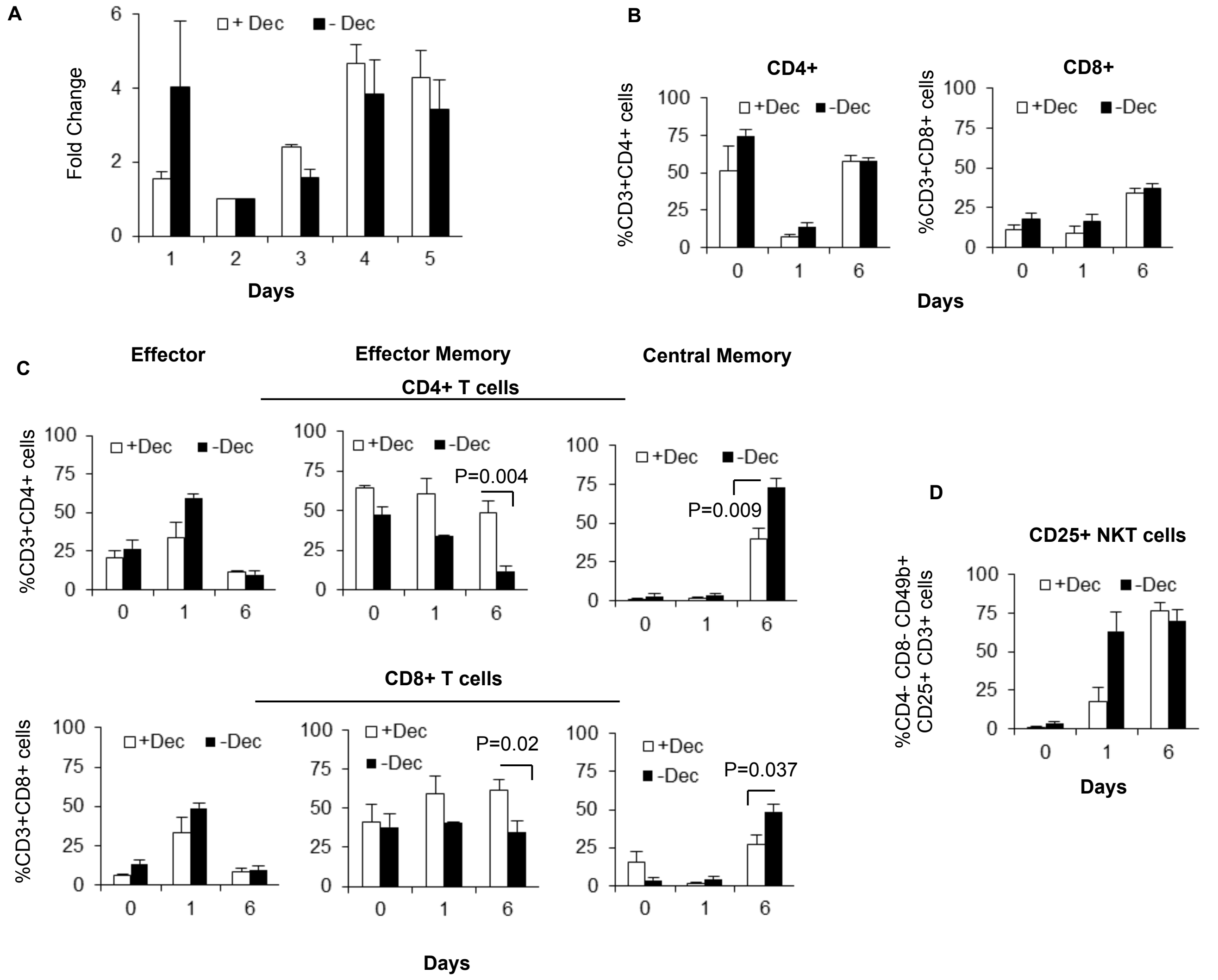

A
+ Dec
- Dec
Fold Change
Days
B
CD4+
CD8+
+Dec
-Dec
%CD3+CD4+ cells
%CD3+CD8+ cells
Days
C
Effector
Effector Memory
Central Memory
CD4+ T cells
P=0.004
P=0.009
CD8+ T cells
P=0.02
P=0.037
Days
D
CD25+ NKT cells
%CD4- CD8- CD49b+ CD25+ CD3+ cells
Days

Supplemental Figure 1. Expansion and phenotypic reprogramming of tumor-reactive splenocytes is similar between animals bearing primary cancer with and without Decitabine preconditioning. FVBN202 mice were challenged with 3 x 10^6 MMC cells intradermally. A portion of the mice went on to receive five sequential injections of Dec (2.5mg/kg) once tumors reached $1000mm^3$ (+Dec), while the remaining mice were untreated (-Dec). Mice were euthanized and spleens were harvested 7 days after the final injection of Dec, and were then treated with B/I and g-c cytokines *ex vivo*. A) Cell counts of viable tumor-reactive immune cells was determined by trypan blue exclusion; fold change was calculated by normalizing the cell count of each day to the number of cells present on day 1. Flow cytometry was used to determine the frequency of total CD4+ and CD8+ T cells (B), phenotype of CD4+ and CD8+ T cells (C), and the frequency of CD25+ NKT cells (D). Data represent four biological repeats for each group and mean ± SEM.

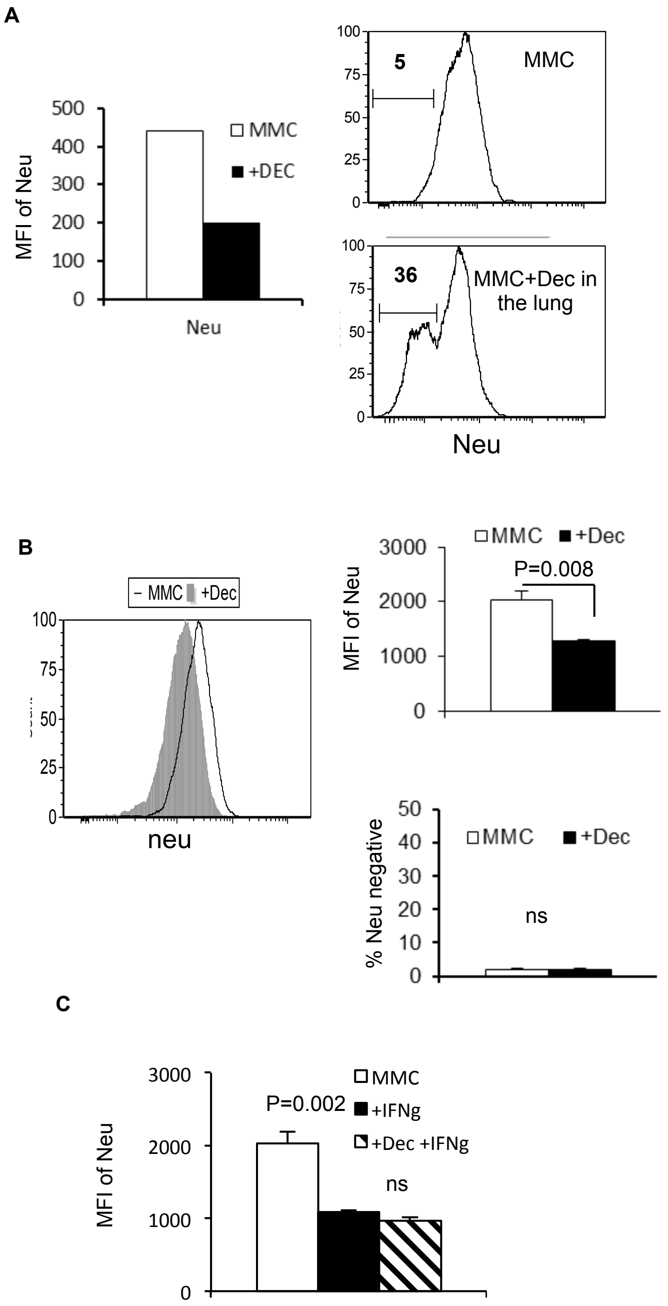

Supplemental Figure 2. AIT promotes immunoediting of lung metastatic lesions. A) FVBN202 mice were challenged i.v. with MMC cells (1 x 10^6); 3 days later they were injected with Dec (2.5mg/kg) once daily for 5 days or remained untreated (MMC). After the mice became moribund, metastases were excised from the lung and established *in vitro*. Neu median fluorescence intensity (MFI) and percentage of neu negative cells were quantified using flow cytometry 10-14 days after the animals had been euthanized. B) MMC cells were treated with Dec (Dec; 3uM) or remained untreated (MMC), *in vitro*. After 10 days of culture, neu expression was quantified using flow cytometry. C) MMC cells remained untreated or were treated with one dose of IFN-γ (50ng/ml) or Dec+IFN-γ, *in vitro*. Expression of neu was determined 7 days after the final treatment. Data represent mean MFI ± SEM of triplicates.

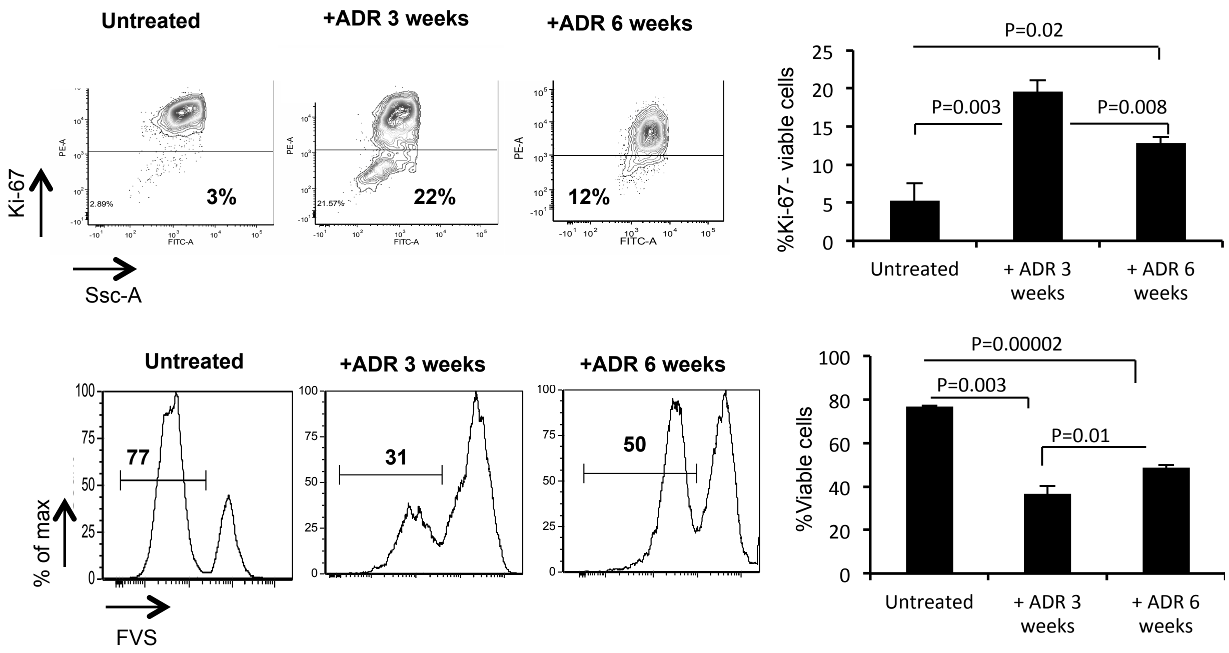

Supplemental Figure 3. Dormant tumor cells recover proliferative capacity as a function of time. MMC tumor cells were treated with 3 daily doses of ADR (1uM for 2 hs), then remained untreated for 3 weeks and 6 weeks, *in vitro*. At weeks 3 and 6 post-treatment, Ki-67 expression and viability were quantified within the population of adherent tumor cells. Data represent 3 independent experiments and mean ± SEM.

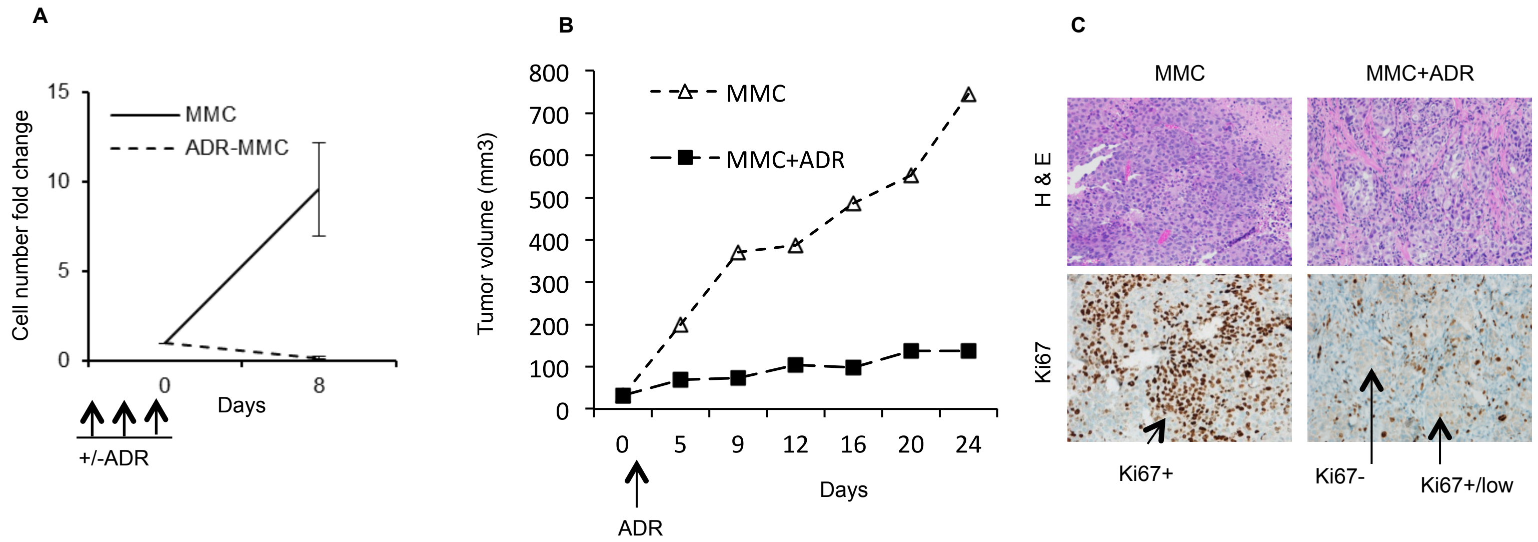

Supplemental Figure 4. ADR treatment induces tumor dormancy. A) MMC cells (n=3) treated with ADR (1uM, 2 hs) for 3 consecutive days and remained in culture for 8 days total, in order to establish tumor cell dormancy, *in vitro*. B) FVBN202 mice were challenged with MMC ($3x10^6$) in the mammary gland region; after tumors reached 30-50 mm^3 animals were treated with ADR (MMC+ADR; 20 mg/kg; i.v.), or remained untreated (MMC). Tumor growth was monitored for four weeks, C) Animals were sacrificed and tumor specimens were collected and subjected to H & E staining as well as IHC for Ki67. Arrows show Ki-67+ proliferating tumor cells (dark brown), Ki-67- quiescent tumor cells (blue color like background) and Ki-67+/low indolent tumor cells (weak brown). Figures show a 200X magnification.
